# Depressive symptoms and suicidal ideation during the COVID-19 pandemic in Russia

**DOI:** 10.1192/j.eurpsy.2021.1758

**Published:** 2021-08-13

**Authors:** T. Medvedeva, S. Enikolopov, O. Boyko, O. Vorontsova

**Affiliations:** Clinical Psychology, Federal State Budgetary Scientific institution “Mental health research center”, Moscow, Russian Federation

**Keywords:** COVID-19, Depression, coping, Suicidal ideation

## Abstract

**Introduction:**

The COVID-19 pandemic may bear serious consequences for mental health, such as the increase in psychopathological symptoms.

**Objectives:**

Analysis of changes in depressive suicidal ideation symptoms and during the COVID-19. Depressive symptoms and suicidality were considered separately.

**Methods:**

Internet survey 22.03.20–22.06.20 (908 responses), included SCL-90R, COPE, question about suicidal ideation.

**Results:**

The analysis showed a positive correlation between suicidal thoughts and depression (Spearman .45; p<.001), a growing trend in the depressive symptoms (Std.J-T=2.51, p=.012), and the increase in severity of suicidal thoughts (Fisher’s Exact Test, 5.92, p=.046). Severity of depression positively correlates with the emergence of the virus in the city, contraction of disease among friends (Spearman .165; p<.001), sick and die fears, an also is linked to more expressed seeking «emotional, social and instrumental help» (COPE). The intensity of suicidal ideation was not associated with these factors, but negatively correlated with «acceptance» and «planning».

**Conclusions:**

The increase in depressive symptoms and suicidal thoughts is determined by different factors. Depressive symptoms is associated with various fears and mediated by non-constructive ways of coping, but there are also constructive coping-strategies as the search for help. The intensity of suicidal thoughts is associated with higher levels of stress, which cannot be explained by the «objective» threat of contagion and fears, but is experienced as an «indefinite» anxiety, supposedly linked to the measures to counter the pandemic, such as restrictions on social interactions, loneliness and uncertainty. The increase in depressive symptoms is linked with an orientation to another person, but the suicidal ideation is not.

**Disclosure:**

No significant relationships.

